# Rapid method for the determination of some organophosphorus insecticides in a small amount of serum in emergency and occupational toxicology cases

**DOI:** 10.4103/0019-5278.55125

**Published:** 2009-08

**Authors:** Bhoopendra Singh, T. D. Dogra

**Affiliations:** Department of Forensic Medicine & Toxicology, All India Institute of Medical Sciences, New Delhi – 110029, India

**Keywords:** Serum analysis, organophosphorus pesticides, gas chromatography with NPD

## Abstract

A simple and rapid method is described for the estimation of some organophosphorus insecticides in the serum of occupationally exposed persons. The compounds are extracted with a mixture of acetone and diethyl ether (1:1 v/v) in acidic medium and the extraction residue is analyzed by gas chromatography with nitrogen phosphorus detection method. Linearity was acceptable over concentrations from 0.25 to 4.0 *μ*g/mL. The method percentile recovery for the six different organophosphorus insecticides was 86.3% for phorate, 78.3% for dimethoate, 82.3% for malathion, 79.4% for chlorpyrifos, 80.2% for diazinon, and 68.5% for ethion at the *μ*g/mL level. Serum samples of nine workers who had been occupationally exposed to malathion in an insecticide manufacturing factory, were analyzed and malathion was found at low levels in all the samples.

## INTRODUCTION

The diagnosis and therapy of poisoning from exposure to organophosphorus insecticides is mostly based on the history and symptomatology of the patient and on the determination of blood cholinesterase activity. For some insecticides, the metabolites are detected in the urine[1,2] and after oral ingestion, the compounds can be identified by the analysis of the gastric lavage.[3] However, in order to manage patients with acute, subacute, and chronic poisoning from these insecticides, it would be of interest to know the fate of the organophosphorus cholinesterase inhibitor and its relationship to the clinical situation. To study this problem, serum or plasma concentrations of the insecticides would have to be determined. Several methods have been described in literature for the determination of organophosphorus insecticide levels in blood, e.g., gas chromatography without sample extraction,[3,4] gas chromatography with sample extraction using an electron capture detector,[5,6] a halogen phosphorus detector,[7] and a nitrogen detector.[8] The use of extraction followed by reversed phase high performance liquid chromatography has also been reported.[9] These methods are typically not sensitive, specific, accurate or rapid enough to allow the emergency toxicologists to determine which of the insecticides are present in the serum, and if they are present, at what levels. In order to address these issues, a rapid, accurate, and sensitive method has been developed for this application and is reported in this manuscript.

## MATERIALS AND METHODS

### Chemicals

**Figure 1:**
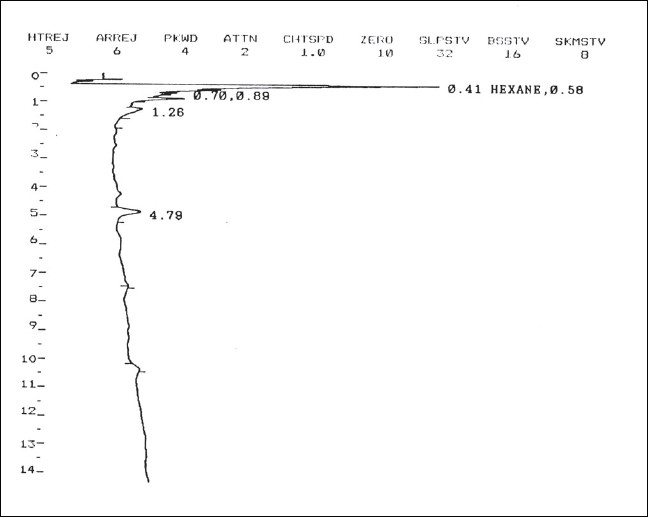
Chromatogram of Blank Serum

Acetone (AR) and diethyl ether (AR) were steam-distilled just before use. Anhydrous sodium sulphate (AR), 5N hydrochloric acid, ν-hexane (HPLC grade) were supplied by E-Merck (India). Pure organophosphorus insecticides (names and structures are reported in Figure 1) were obtained from Supelco Sigma Company (USA).

### Standard Solutions

Twenty millilitres of standard stock solutions of phorate, dimethoate, malathion, chlorpyriphos, diazinon, and ethion were prepared at a concentration of 1 mg/mL in acetone (HPLC Grade). Working solutions were prepared in ν-hexane by appropriate dilution of the standard stock solutions to obtain the desired concentrations of these organophosphate compounds in the range of 0.25 to 4.0 *μ*g/mL. A 4 *μ*g/mL working solution of the internal standard (Diazinon) was prepared in ν-hexane. These solutions were stored at −20°C and found to be stable for at least three months. Solutions of individual compounds were injected into the chromatograph to determine the retention times of the insecticide studied.

### Gas Chromatography

GC analyses were performed on a Nucon Chromatography-Nitrogen Phosphorus Detector 5700 instrument (Nucon Engineer's, New Delhi, India) fitted with a glass column (4 mm i.d, and 1.2 meter length, packed inhouse with 10% of SG-30, mesh range 30–100 on a CHW-PW support). The carrier gas used was high-purity nitrogen at a flow rate of 60 mL/min (1 kg/cm^2^ head pressure). The fuel gases used for the NP detector were hydrogen and air (zero) at flow rates of 5 mL/min (0.8 kg/cm^2^) and 80 mL/min (0.8 kg/cm^2^) respectively. The initial oven temperature was set at 180°C for 1 min, then increased at the rate of 6°C/min to 250°C and held there for two minutes. The injection port and detector temperatures were 260°C and 280°C respectively. All compounds, including the internal standard, were eluted within 15 min.

Spiked serum samples and calibrators: To prepare the serum samples for setting up the extraction conditions and for quantification of the target analytes, aliquots of a pool of blood sera from local, healthy, pesticide-naïve donors were spiked with 0.50 to 4.0 *μ*g/mL.

### Extraction and Preparation of the Sample:

One milliliter of serum was taken in a 10 mL glass stoppered tube to which was added 4 mL of a mixture of acetone and diethyl ether (1:1 v/v). The mixture was vigorously shaken for five minutes using a cyclomixer and acidified with 0.2 mL of 5N HCl. The mixture was again shaken for a minute, taking the caution of avoiding prolonged contact of the sample with the 5N HCl solution to prevent the decomposition of the compounds. The organic layer was separated with a pipette and transferred to a separate tube. The residual slurry was again extracted two more times with 4 mL of diethyl ether. All supernatants, for a total volume of approx. 10mL of organic solvents, were combined in a separate beaker (25 mL), and passed through 2 g of anhydrous sodium sulphate to remove inorganic phosphate and water contents. The organic filtrate fraction was evaporated to dryness under a gentle stream of nitrogen gas. The residue was dissolved in 0.2 mL of ν-hexane and a 1-*μ*L aliquot was injected into the gas chromatograph.

Method validation: Organophosphate-free serum samples obtained from healthy volunteer subjects were used as negative quality control samples (NQC). For the determination of the limit of detection (LOD), NQC serum was spiked with containing 0.25, 0.50, 1.0, 2.0, 3.0, and 4.0 *μ*g/mL of the analytes.

Selectivity: Each blank sample was tested for potential interferences to analyte concentrations at the lower limit of quantification.

Accuracy: The accuracy of the method was tested by repeated analyses of samples that contained known amounts of the analyte of interest, and it is reported as the deviation of the mean from the true value.

Calibration curve: Generated for every analyte by covering the expected range of 0.25 to 4.0 *μ*g/mL.

Assay Recovery: Recovery experiments are performed at three concentrations (low: 0.50 *μ*g/mL, medium: 2.0 *μ*g/mL, and high: 4.0 *μ*g/mL) by comparing the results for extracted samples with unextracted reference standards.

Patient's samples: Serum samples of nine persons employed in a pesticide manufacturing unit were analyzed. The samples were stored at −4°C before extraction and analysis.

## RESULTS AND DISCUSSION

**Figure 2:**
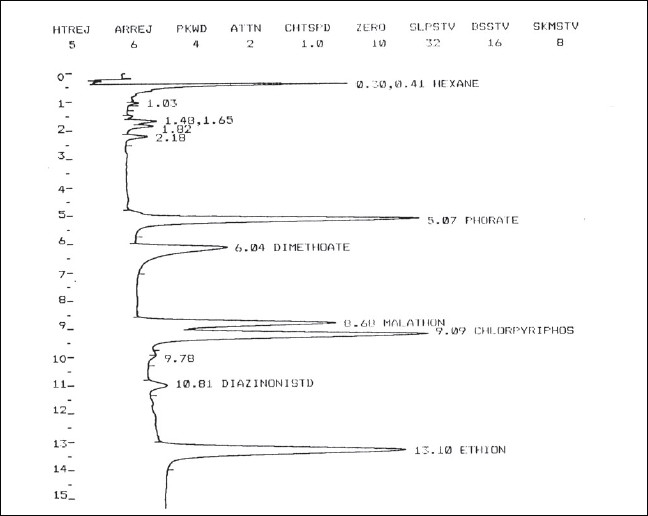
Chromatogram of test mixture containing six organophosphate insecticides

Figure 1 shows the gas chromatographic analysis of a test mixture of the six insecticides in ν-hexane, each at a concentration of 3.0 *μ*g/mL. The gas chromatographic conditions were chosen so that the analysis was complete in 15 minutes and the compounds were resolved well, even the closely eluting Malathion and Chlorpyriphos. The Nitrogen Phosphorus Detector contains a temperature-controlled, electrically heated alkali metal source which remains stable with flow change and protects the alkali metal source from overheating. It offers a high sensitivity and selectivity to nitrogen- and phosphorus-containing organic compounds without the use of an expensive mass-selective detector. The retention times of the five target insecticide analytes and of the internal standard Diazinon are reported in Table 1. The differences in retention times were observed at various concentrations ranging from 0.25 to 4.0 *μ*g/mL of the pure standard and in spiked serum extracts. As apparent, the relative retention times (RRT) of the five analytes with reference to that of the internal standard are sufficiently stable as to allow a reliable identification of the analytes in real-life samples [Figure 2].

**Table 1:** Retention times (RT) and Relative Retention Time (RRT) of the analyzed pesticides

Pesticides	RT (Min)	RRT
Phorate	5.52 ± 0.170	0.490
Diamethoate	6.45 ± 0.250	0.570
Malathion	9.16 ± 0.010	0.810
Chlorpyriphos	9.62 ± 0.020	0.850
Diazinone (I.S.)	11.31 ± 0.060	1.000
Ethion	13.65 ± 0.060	1.210

Mean of 5 injections of pesticide mixtures extracted from serum at concentrations 0.50, 1.0, 2.0, and 4.0 *μ*g/mL. Calculated with reference to the mean RT of the internal standard (IS) in all injections.

The turnaround time for extraction and analysis is 1–2 hr, including the run of the appropriate comparison standards for pesticide identifications and of calibrators for quantification.

The extraction recoveries from serum are listed in Table 1.

In the present study, hydrochloric acid was used in the extraction of pesticides to optimize the percent recovery. The hydrochloric acid helped to improve analyte recovery from the serum samples [Table 2]. Similar results were also showed by others in the analysis of abate,[10] Chlorpyriphos-methyl,[11] and 13 organophosphorus pesticide compounds.[12]

**Table 2:** Recovery (%) of the analyzed pesticides from acidified and nonacidified spiked serum extracted with organic solvent (see text)

Pesticides	HCl Addition	Without HCl Addition
Phorate	83.5 ± 0.300	64.2 ± 0.249
Diamethoate	89.2 ± 0.404	75.9 ± 0.300
Malathion	95.3 ± 0.404	78.4 ± 0.451
Chlorpyriphos	88.7 ± 0.200	77.3 ± 0.458
Ethion	75.8 ± 0.351	63.3 ± 0.404

Mean of 3 injections of pesticide mixtures extracted from serum at concentrations 0.50, 1.0, 2.0, and 4.0 *μ*g/mL

For quantitative analysis, a calibration curve was generated for each insecticide for concentrations from 0.25 to 4.0 *μ*g/mL by running the same sample thrice. An average of these values was used to calculate the average percentage recovery of the pesticides indicated in Table 2. Calibration curves for the five target analytes were calculated by plotting the peak area of sample *vs*. amount of the sample injected. The correlation coefficients of the calibration curves were 0.999 for Phorate, 1.000 for Diamethoate, 1.000 for Malathion, 1.000 for Chlorpyriphos, and 0.997 for Ethion. Hence, quantitative analysis can be carried out in this range.

Serum samples were obtained from nine employees working in a pesticide manufacturing unit and tested using the developed method. The only insecticide found to be present in the samples was malathion at levels of 0.5–0.8 *μ*g/mL.

A simple and rapid extraction procedure has been described for the quantitation of organophosphorus insecticides in the serum of poisoned patients. The use of this method may facilitate the diagnosis and management of cases of poisoning by these insecticides.
